# Small ruminants as a pathway to reduce urban poverty: An empirical analysis of Sudan

**DOI:** 10.14202/vetworld.2019.2017-2024

**Published:** 2019-12-20

**Authors:** Raga Elzaki, Samar Abdalla, Mohammed Al-Mahish

**Affiliations:** 1 Department of Agribusiness and Consumer Science, College of Agriculture and Food Science, King Faisal University, Al-Ahsa 31982, Saudi Arabia; 2 Department of Rural Economics and Development, Faculty of Animal Production, University of Gezira, Sudan; 3 Agricultural Research Corporation, Agricultural Economics and Policy Research Centre, Shambat, Sudan

**Keywords:** goats, sheep, urban poverty causes, urban poverty line, urban poverty reduction

## Abstract

**Aim:**

This study aimed to measure the energetic incidence of poverty and determines the main factors that cause urban poverty. Moreover, the study examines the key role of the livestock sector in poverty reduction in urban regions and develops an analytical tool to aid in urban area poverty mitigation through goats and sheep ownership.

**Materials and Methods:**

The study mainly depends on primary data assembled through structured and unstructured questionnaires, which were distributed among the targeted groups in the urban area in Sudan. Poverty line and poverty indices were calculated and measured using various well-known methods. The causes of poverty were estimated using logistic regression, and the effect of small ruminants in poverty alleviation was estimated using multivariate regression analysis.

**Results:**

The study findings indicate that both food and income poverty lines are less than the standard poverty line. In addition, the results imply that rural migration and crime predictors are among the most important factors in increasing urban poverty in the study area. Furthermore, livestock ownership has a significant impact on poverty reduction.

**Conclusion:**

The study concludes that small ruminants are playing a key role in reducing urban poverty. Thus, the study urges planners and policy-makers to support policies that promote livestock sector development as a strategy to alleviate poverty in Sudan.

## Introduction

Sudan is an agrarian developing country classified as low income with a per capita income of <$1,045 (USD) per year (2019). The Gross Domestic Product at constant prices in 2016 was 41.3 [1]. The last National Household Budget and Poverty Survey in Sudan conducted by the Central Bureau of Statistics (CBS), during 2014/2015 confirmed that 36.1% of the population in Sudan was living in absolute poverty while urban poverty accounted for 39.9%. The per capita/year is 2966 Sudanese Pound (SDG) in urban areas, which displays higher average consumption levels than rural areas (SDG 2698). Poverty assessment in Sudan has been limited, but studies have provided evidence of high-income poverty. A study conducted by Faki [2] analyzed poverty with wide coverage by state and based on aggregate consumption of five main components (food, non-food, durable goods, housing, and energy). It puts North Sudan at an overall poverty level of 46.5%. Rural poverty was way above urban poverty (27%). An authorized study performed by CBS [1] conducted all over Sudan regions reported that the poor population in urban areas is slightly higher than in rural areas.

Livestock production is a dynamic sector for the National Economy in Sudan. Sudan total domestic livestock population in 2017 was about 104 million heads. Goat populations constituted more than 31 million heads, while sheep population constituted nearly 40 million heads [3]. At the national level, the livestock sector is characterized as the most active sector in national income. The livestock shares are estimated to be 18-25%, and it shares foreign currencies through the export of the various livestock products in the form of live animals, meat, and leathers. In addition, it represents a livelihood activity for about 60% of the population and provides employment for about 40% of the population [4]. At household level, livestock is the main source of food, employment, income, transportation, prosperity, and enhancing crop production by providing organic manure fertilizers and draught power. Shrestha *et al*. [5] indicated that when crops are not sufficient to ensure food security, livestock can be used as a source of food.

Nowadays, in Sudan, no adequate studies have investigated urban poverty reduction, whereas a majority of international, national, and regional research has focused on rural poverty reduction [6-9]. The lack of updates to the poverty line and studies of economic policy in urban areas have made it difficult to make comparisons across time, particularly in recent years, through which the country’s economy has deteriorated and marginally fallen. Moreover, no recent research has been accomplished toward poverty reduction in Sudan. Likewise, civil wars and political problems led to successive migration from unsecured areas, particularly from rural areas, to urban areas. The unplanned migration created various and serious problems faced by the migrators. Hence, most of the migrators have no access to the basic needs of life and struggle to live in a new environment. Furthermore, the abrupt financial fall of the economy successively led to increased poverty among the rural migrators; those migrators reverse their initial wealth, which are mainly seasonal crops and rearing of livestock.

This study aimed to analyze and measure the poverty indices in the urban areas and to identify the main factors beyond urban poverty causes. Furthermore, the study attempts to construct an identical strategy approach of urban poverty reduction by practicing small ruminants in the indoor household.

## Materials and Methods

### Ethical approval

For this study, the collection of the questionnaire was approved by the Faculty of Animal Production, University of Gezira, Sudan. The oral permission was obtained from the target’s household head in the study area.

### Methodology and data description

Bahri region is selected to act as the study area (located in the Eastern part of Khartoum National Capital). It was selected because the majority of livestock is distributed in this region compared to other regions around Sudan capital. The study mainly depends on primary data assembled through structured and unstructured questionnaires which were distributed among the targeted groups in the urban area in Sudan. About 300 questionnaires were distributed randomly for the household heads in the Bahri region (year 2017/2018). As the study mainly concentrated on extreme poverty (food and non-food expenditures) in the region, the primary data collected to cover the information of monetary (economics data) and nonmonetary indicators (demographic and socioeconomic). The major data include household head ages, jobs, family size, income, foodstuff (types, purchasing prices, consumption, etc.), health, education, housing conditions, water aspects, and its problems, social participation, political conflicts, crimes, and various features of livestock issues.

### Poverty line and measurement method

The poverty line is calculated based on dietary intake kilocalories per equivalent, which is later termed as food poverty, and the situation of household income and expenditures is termed as money or income poverty.

Poverty is a complex and multifaceted problem, and varieties of methodologies are used for poverty calculation and examination. According to objectives and the nature of the data collected, poverty was calculated using the Foster-Greer-Thorbecke (FGT) method suggested by Foster *et al*. [10]. The investigation follows the cost of basic needs method to poverty analysis, which was performed in many African countries. The three sound methods of poverty measures (headcount ration, poverty gap, and poverty square) were analyzed by succeeding FGT method. In addition, a fourth measure (Watts’s index) was calculated. Watts’s index takes into account both income and the number of people in poverty [11].

### Poverty line

Assuming that the *Y_i_* denotes household income and *Z* denotes the poverty line, both income and poverty line were calculated by comparing the total expenditure of the individual household by the international poverty line, which was originally set to be roughly <$2/day [12]. In addition, poverty line was calculated by summation of the kilocalories of Sudanese food staff to the standard kilocalories requirement according to the World Health Organization. The minimum level of Sudanese household’s expenditures should enable them to buy 2100 kcal of food per person per day [13]. If the individual consumes less than the required kilocalories (2100), it was considered poor; otherwise, it was non-poor. Poor = 1 if *C* ≤ 2100 and non-poor = 0 if *C* > 2100, whereas the *C* = total consumption of kilocalories per day. For estimating the number of poor and non-poor according to their daily expenditure in USD, we assign the value 1 when income (*y_i_*) falls below the poverty line (*z*) and 0 if income fall above the poverty line. Thus, poor = 1 if *y_i_* ≤ *z* and non-poor = 0 if *y_i_* > *z*.

### Poverty indices

The general FGT equation of poverty indices was measured as below:


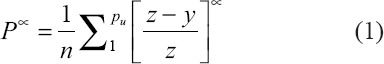


### The headcount

The headcount was calculated by matching the *Y_i_* of each household to *Z*. The headcount index (*H_i_*) was the sample average of the variables weighted by the number of people in each household *n_i_*. The parameter *α* determines the measure sensitivity to the degree of deprivation for those below the poverty line [10]. When *α* equals zero the measure reduces to the below formula:


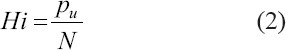


### Poverty gap (depth)

The poverty gap of urban people represents the depth of poverty. It means the distance separating the people from the poverty line. It was generated when the parameter *α* equals *s* one, which can be defined as follows:


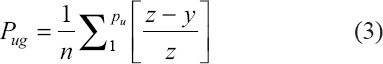


Whereas *P_ug_* is the urban poverty gap.

### Poverty square (severity)

Poverty square or severity reflects the inequality between the poor household. This measure is illustrated by the below equation:


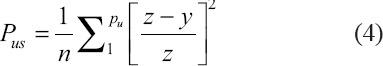


### Watts’ index

The study tries to calculate Watts’ index, which is a function of an individual income and poverty line. It is the first distribution-sensitive poverty measure that was proposed by Watts [11]. Watts’ index is calculated by dividing the poverty line by income of the urban sample of the adult equivalent [14]. Taking logs, (which involves the computation of the logarithm of each income) and taking the sum over the poor and the household size variable *n_i_* replaced with the adult equivalent size *a^i^*. According to World Bank [15], the equation of Watts index is represented below:


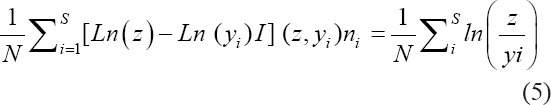


Where *s* is the total number of the household in the sample.

### Logistic method of urban poverty causes

The study follows various researchers [16-20] to estimate poverty causes using binary logistic regression. Binary regression is used to predict the relationship between dependent variables (predicted variable), which is dichotomous and represents poor versus non-poor households. The independent variables (predictors) represent the demographic features of the sampled population and the types of livestock.

To identify key determinants or causes of poverty, a dichotomous variable indicating whether the household is poor or not is computed [19]. Estimated probability of being poor, given the values of explanatory variables, in this study is a single categorical variable:


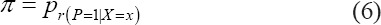


*π* = is predicted probability, *P_r_* = probability, and *P* is a binary variable of poor household. *P_i_* = 1 if the household is poor in observation *i*, otherwise *P_i_* = 0, if the household is not poor in observation *i*.

The *X_s_* values are a set of predictors, which can be discrete, for instance, number of animals, family size, etc., also, it can be continuous, such as age and income.

Later the poverty binary model could be:


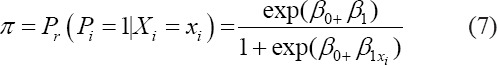



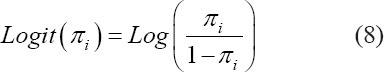



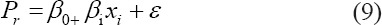


*β*_0_ = a cluster fixed or random effect and *β* is a vector of parameters.

*x_i_* is a vector of household demographic features or others explanatory variables, which include a set of individual characteristics *X* (e.g., gender, education, age of the household head, and livestock ownership). Furthermore, some political variables are included in the model, for example, rural migration due to crimes and/or conflicts.

### Poverty reduction model

One of the key issues of various international institutions [21,22] is to suggest and adopt the various types of policies and strategies for poverty reduction in the world in general and poverty reduction in developing countries in Africa, specifically. Hence, widespread malnutrition and most African nations are agrarian and depend mainly on crop production as the main source of income generation. The strategy used for urban poverty reduction in this study is through livestock practicing. The study selected two livestock, which is goats and sheep. Thus, these two types of livestock are much cheaper and simply can be kept inside homes.

### Poverty line elasticity model

Numerous researchers investigate poverty reduction, taking into account the different forms and formula of poverty elasticity with respect to growth, income, inequality, and standard of living [10,23-26]. Our study tries to analyze the poverty lines’ (income and food poverty) elasticities to display the responsiveness, or how poverty lines change in response to a livestock production change.

To explain the role of livestock in poverty eradication in urban areas, the study uses a regression model to evaluate the incorporation of the livestock in the household to reveal the importance of practicing livestock activities in the household. Multiple regression procedures are constructed to explain the relative response of per capita income to increases in goats and sheep heads, and hence the poverty alleviation. The equation can be written as follows:


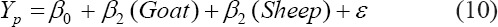


Equation (11) is a log-linear model aiming to estimate the poverty elasticity with respect to numbers of sheep and goats owned by the household as follows:


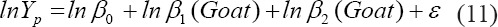


Where *Y_p_* = poor income and the predictor variables are goat and sheep heads, respectively. Extra equations of the multiple regressions are performed to show the effect of livestock production in food poverty reduction. In this equation, the dependent variable used is food poverty line and the independent’s variables used are consumption of both milk and meat in the households, as shown by Equation (12).


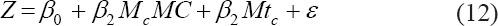


Equation (13) is a log-linear model to estimate poverty elasticity with respect to milk and meat consumption by the household as follows:


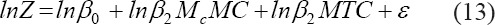


Whereas *Z* = food poverty line, *MC* = milk consumption, and *MTC* = meat consumption. The regression models were executed using the capabilities of SPSS, SAS, and Excel software programs.

## Results

The descriptive results show that 65% of respondents are males while 35% are females. The majority of females migrated from civil wars or conflict areas in Western or Eastern regions or escaped from natural hazards in Northern regions.

The average age of the surveyed household headed is 52 years. The average family size is found to be approximately eight people, and this is a normal phenomenon in Sudan.

Considerable numbers of the surveyed samples (35%) are unschooled and almost illiterate, and practicing the informal jobs ranged between street sellers to hired labors with marginal employers. Only 4.3% received university education and those were the only segment of the sampled survey who own houses. According to the food poverty line (household member that consumed (<2100 kcal), the numbers of the poor constituted nearly 222 (74%) while the non-poor are 78 (26%). Most of the poor households are females (85%) and the remainder are males (15%).

### Urban food consumption and poverty line

From Table-1 [27], it is clear that household actual consumption is 1888.09 cal, which is less than the recommended calories by 9.6%. It is notable that coffee comprises a higher food poverty line (SDG 107.96) followed by cereal food (SDG 62.35). In addition, the study result confirms that the food poverty line is 8.48/household and equal to $1.04/person (average family size eight people). The non-food expenditure is estimated to be 132.5 SDG. Furthermore, the extreme poverty line is estimated to equal nearly 11.80 SDG/household and $^1^1.47/person (Tables-1 and 2).

**Table-1:** Food consumption of households in Bahri region.

Food elements	ACC/day	RC/day*	NC/kg*	P/kg in SDG	Poverty line in SDG
Cereal food consumption					
Sorghum	1178.23	884.1454	0.263924	165.451	43.66
Wheat	52.15	199.4372	0.054941	170.909	9.39
Millet	5.58	194.3744	0.058022	160.383	9.3
Subtotal	1235.96	1277.957	0.376887	496.743	62.35
Animal products food consumption					
Meat	99.23	98.88078	0.048951	250.278	12.25
Milk	32.78	74.15888	0.115873	137.982	15.98
Chicken	23.5	418.9922	0.046555	412.480	19.2
Egg	39.011	6.20615	0.004433	438.950	1.95
Subtotal	194.521	598.238	0.215812	1239.69	49.38
Vegetables					
Okra	7.5	8.946715	0.008284	1950.506	16.15
Onion	12.5	7.52132	0.015669	90.200	1.41
Tomatoes	25.23	29.82238	0.06213	180.653	11.22
Other vegetables	27.21	48.00952	0.032006	780.235	24.97
Subtotal	72.44	94.29994	0.118089	3001.594	53.75
Coffee					
Sugar	177.269	295.7592	0.07394	150.28	11.11
Tea	3.5	11.55864	0.010702	9050.102	96.85
Subtotal	180.769	307.3178	0.084642	9200.382	107.96
Others needs					
Salt	4.15	3.018763	0.013722	850.460	11.67
Oil	200.25	8.583058	0.012089	192.180	2.32
Subtotal	204.4	11.60182	0.025811	1042.64	13.99
Overall total	1888.09	2289.414606	0.821241	5852.049	287.43

Source: Field surveyed results, 2017/2018.

* Data from the World Health Organization [27]. ACC=Actual consumed kilocalories, RC=Required kilocalories, NC=Numbers of kilocalories in food items, P=Price of food items

**Table-2:** Urban poverty measurements in Bahri region.

Income poverty line	Value	Poor and poverty measures	Value
Average of food expenditures	5852.049 SDG	Number of the total samples	300
Average of non-food expenditures	132.5 SDG	Number of poor	222
Total expenditure	5984.549 SDG	Headcount index	74%
Average of family size	Eight persons	Poverty gap (depth)	68.28%
Poverty line	1.04$	Poverty square (severity)	45.26%
Extreme poverty line	1.47$	Watts index	55.25%

Source: Field surveyed results, 2017/2018

As shown in Table-2, the urban poverty measurements reveal that the proportion of those who live below the poverty line is 74%, which indicates that more than half the surveyed sample is poor. Likewise, the poverty depth and severity are estimated to be 68.28% and 45.26%, respectively. Furthermore, it implies that 68.28% of the poor are slightly far from the poverty line (1.04). In addition, the mean proportionate poverty gap of the urban poor is high, as shown by Watts’ index (55.25%).

### Urban poverty causes

The connection between risk factors and the incidence of urban poverty is shown in Table-3.

**Table-3:** Model of risk factors causing urban poverty.

Predictors/explanatory variables	Estimated coefficient (β)	Standard error	Wald	Odds ratio exp (β)	95% of C.I. for odds ration	

					Lower	Upper
Gender	−0.768	0.525	0.856	0.626	0.325	1.700
MHHA	−0.008	0.051	0.120	0.995	0.852	1.053
EDL	−0.358	0.133	2.566	0.460	0.453	1.009
OL	−0.452	0.199	3.926	0.730	0.505	1.078
FIJ	0.003	0.078	0.000	1.002	0.728	2.302
FS	0.778	0.235	0.812	1.512	0.122	1.598
NM	0.815	0.485	1.054	2.052	0.429	8.582
NF	0.528	0.480	1.125	2.058	0.752	6.458
CA	1.221	0.245	11.458	3.316	0.155	0.482
DA	0.296	0.456	0.350	1.344	0.250	5.122
CW	−0.128	0.223	0.268	0.211	0.284	2.596
HC	−0.259	0.487	0.278	1.457	0294	3.256
RMIG	0.810	0.235	9.125	4.125	0.258	6.289
Constant	3.889	1.449	6.758	52.033	χ^2^ = 39.58	

*Note: MAHH=Male household headed age, EDL=Education level, OL=Own livestock, FIJ=Types of jobs, FS=Family size, NM=Numbers of males, NF=Number of females, CA=Crimes attach, DA=Diseases affections, CW=Clean water, HC=Health care, and RMIG=Migration. Binary logistic statistics: *Number of observations=300, Adjusted R-squared: 0.400, 2-Log likelihood=250.613. Source: Field surveyed results, 2017/2018

The goodness of fit of the model is shown to be significant (χ^2^ = 39.58). The logistic regression result shows that there is an increase in the likelihood of being poor with job types, family size, number of males, crimes, diseases, affection, and migration (the odd ratios >1). Male household head age, education level, and livestock ownership have odds ratios <1, which indicate that the likelihood of being poor is decreased for these variables. Education level has a low value of odds ratio (0.460); this indicates that the poor living in urban regions have more access to education. Moreover, the findings show that households with access to clean water were significantly less likely to be in poverty compared to households without access to clean water. However, residents living in extreme poverty suffer from the risks of other factors, such as no access to health care and disease infections (including malnutrition).

Migration and crime predictors were among the most important factors in increasing urban poverty in this study.

### Role of small ruminants in urban poverty reduction

Ram [26], using the panel data of Vietnam, suggested that livestock production contributes to poverty reduction. The multiple regression models were run to estimate the impact of livestock on poverty reduction (Tables-4 and 5). The overall model is significant (F=59.64, R^2^=0.570).

**Table-4:** Influence of small ruminants on per capita income of the urban poor.

Model	Value of coefficient	t-value	Significance	F-value	R^2^
Constant	−1179.04	−1.459	0.148	59.64 (0.000)	0.570
Goats	33.793	3.162	0.002		
Sheep	41.575	5.613	0.000		

**Poor income elasticity**					

**Elasticity**	**DF**	**Parameter estimate**	**Standard error**	**t value**	**Pr > |t|**

Goats	1	0.54095	0.03305	16.37	<0.0001
Sheep	1	0.30192	0.03666	8.24	<0.0001

Source: Fled surveyed results, 2017/2018

**Table-5:** Influence of small ruminants on the total kilocalories consumption.

Model	Value of coefficient	t-value	Significance	F-value	R^2^
Constant	2265.090	2.975	0.003	16.78 (0.000)	0.190
Milk consumption	5.100	0.800	0.425		
Meat consumption	125.916	3.140	0.002		

**Total kilocalories consumption elasticity**					

**Elasticity**	**DF**	**Parameter estimate**	**Standard error**	**t-value**	**Pr > |t|**

Milk consumption	1	0.12644	0.08907	1.42	0.1568
Meat consumption	1	0.95737	0.08391	11.41	<0.0001

Source: Filed surveyed results, 2017/2018

Based on the multiple regression model results, the study shows that the increase in goat numbers lead to increase in the per capita income of the urban poor by 33.79 SDG. In addition, when the sheep numbers increase by one head, the per capita income increases by 41.57 SDG (t=5.613). Furthermore, a 1% increase in goat and sheep heads increases per capita income by 0.54% and 0.30%, respectively.

On the other hand, the results in Table-5 show a direct connection between poverty reduction and consumption of livestock products (meat). If meat consumption increases by 1 kg, the kilocalories of the household increase by 126 kcal. However, milk consumption had an insignificant effect on households’ kilocalories.

Based on the elasticities estimation, all results were inelastic, as shown in Tables-4 and 5. Table-5 shows that a 1% increase in meat consumption results in a 0.957% increase in the urban poor kilocalories.

## Discussion

Urban poverty becomes core attention of the developing countries’ governments within which the people suffer from refugees, civil wars, and resource conflict. Adequately, studies are performed worldwide using different methods of urban poverty measurements and roughly compare rural poverty to urban poverty.

In this study, the results show that there is an increase in the likelihood of being poor with family size (Table-3) and this result confirmed by Kabir and Maitrot [28] who stated that the larger the size of the beneficiary’s household, the more negative the effect on economic growth and well-being. Furthermore, this study reveals that migration is constantly contributing to increase poverty incidence in the urban area; however, Christiaensen [29] showed that in Tanzania, the migration to secondary towns helps in poverty reduction.

In addition, De Janvry and Sadoulet [30] analyzed the change in the relative number of rural and urban poor using aggregate analysis. Their findings showed that the incidence of rural poverty was declining relative to the incidence of urban poverty, and the population was rapidly leaving in the rural sector. In addition to the universal increase in commodity prices, food prices steered to increase urban poverty incidences. The study results indicate that food price is high, which is influenced by urban poverty and this result agrees with the findings argued by Meng *et al*. [31] that the poverty line is linked positively with an increase in relative food price. Study shows a deep level of deprivation and higher incidence of poverty for urban people who are under the poverty line in the study area, while De Janvry and Sadoulet [30] found that urban poverty dominates aggregate poverty; the urban poor captures more than half of the aggregate increase in real income. Furthermore, De Janvry and Sadoulet [30] indicated that the incidence of rural poverty in Latin American countries is considerably higher and deeper than the incidence of urban poverty. The study result displays that the poverty income is sensitive in the urban area; however, De Janvry and Sadoulet [30] stated that the overall rural poverty is less sensitive to aggregate income growth compared to urban poverty and Yamada [32] used the quantile regression model and argued that the coefficients in urban areas are larger than rural area. Moreover, the regression coefficients have been decreasing slightly as time passes and do not have constant changes across the deciles. Employment tends to affect food poverty dynamics differently in urban and rural areas. The results of logistic regression used in this study reveal that there is an increase in the likelihood of being poor with job types. Eigbiremolen and Ogbuabor [33] used the dynamic food poverty continuous model in Nigeria to examine the impact of selected covariates on (log) growth of food consumption expenditure and the outcome showed that urban households which have a household head that is employed, increases his/her per capita spending on food over time in relation to households whose heads are unemployed. Thus, the authors conclude that employment reduces food poverty. Cho [34] indicated that households in urban areas were found to be multi-dimensionally poor. Some researchers went beyond child labor poverty in urban areas, such as Dayioğlu [35], who investigated the determinants of child labor in urban Turkey with low household income or in poverty using a probit model. The results showed that child employment goes down with household income. However, the effect of household income on child employment is not great, and the likelihood of children’s employment is highly significant.

Poverty reduction has been a political priority from most developing nations and received great attention to international organization agendas [26,36]. The strategy used for urban poverty reduction in this study is through livestock practicing. Accordingly, different strategies and policies of poverty reduction are executed worldwide. Alwang *et al*. [37] examined the poverty reduction through innovations to improve staple crop germplasm and found that the main difficulties to greater poverty reduction include limited access to credit, services and markets, and small landholding sizes of poor farmers. Furthermore, the authors confirm that landholding size is an important barrier to poverty reduction. In addition, farm’s technologies that enhance farm productivity can be a sustainable pathway to improve household food security and to enable households to climb out of poverty [38]. However, van Noordwijk [39] examined integrated natural resources management as a pathway to poverty reduction, while Benfica *et al*. [40] argued that providing extension services to smallholders is most effective at raising growth and reducing poverty. An increase in the output would increase farmers’ income and reduce poverty in rural areas [41].

The farmers adopting more adaptation practices have higher food security and a lower level of poverty [42]. Martin [43] argued that the focus should be on the sustainability of local socio-technical systems, even if the options chosen are less efficient in the short term. Hansen [44] argued that climate-risk management interventions could play in efforts to reduce rural poverty. There are no studies and/or reviews that empirically analyze the relationship between poverty reduction and women’s human rights [45].

The study results confirmed that urban poverty could be alleviated and reduced by the consumption of livestock products (Table-5). By viewing the alleviation and reduction of poverty through practicing livestock, remarkable investigations have performed in this matter. Do *et al*. [46] evaluated the impact of livestock production on poverty reduction and estimated the average treatment effect on the treated using the matching-difference-in-difference method. They indicated that owning a large livestock size meaningfully reduces the depth of poverty, and access to dairy livestock assets can provide important benefits for women [36]. However, Ali [47] argued that despite climate risk, decreased livestock production increased household income and lowered poverty levels.

## Conclusion

Most developing countries face both rural and urban poverty during recent years. Based on the case study in urban Sudan, this article measures the incidence of poverty and determines the main factors that cause urban poverty. Furthermore, the study examines the vital share of the livestock sector in urban poverty reduction and develops an analytical tool helping to mitigate poverty in urban areas through goats and sheep ownership. The study adopted the standard tool of poverty measurements and estimated the urban poverty line and poverty indices (headcount, depth, severity, and Watts index). Furthermore, the study used logistic regression to reveal the likelihood of poverty determinants. Multiple regression models were also used to show the role of livestock in poverty reduction. In addition, livestock elasticities were estimated to display the responsiveness of poverty lines to changes in livestock production.

The model incorporated small ruminants as a strategy for poverty alleviation. The results indicate that both food and income poverty lines are less than the standard poverty line. In addition, the results implied that rural migration and crimes are among the most important factors contributing to the increase in urban poverty in the study area. The study concluded that small ruminants are playing a key role in urban poverty reduction. The study suggests that planners and policy-makers should encourage policies that promote livestock development and increase financial credit of livestock production. The purpose is to escalate the urban poor beyond poverty and helps them to obtain the basic needs of life, especially for the migrators who escaped from vulnerable regions.

## Authors’ Contributions

RE suggested and scheduled the study, managed the data collection, analyzed and manipulated the statistical analysis, and interpreted the results, and approved the final draft of the manuscript. MA contributed to data analysis and interpretation, modified, and revised the final draft of the manuscript. SA wrote the literature review and approved the final draft.
